# A Nearly Missed Pancoast Tumour From Isolated Persistent Leg Pain

**DOI:** 10.7759/cureus.15328

**Published:** 2021-05-30

**Authors:** Moussa Issa, Zain ul Abadin

**Affiliations:** 1 Emergency Medicine Department, Midland Regional Hospital at Tullamore, Tullamore, IRL; 2 Emergency Department, St. James Hospital, Dublin, IRL

**Keywords:** pancoast tumour, superior sulcus tumour, leg pain, emergency department, persistent leg pain, metastatic leg lesion

## Abstract

Pancoast tumours (PTs) are apical tumours of the lung that manifest with a variety of symptoms. Herein, we describe a rare case of a 56-year-old female with a one-month history of persistent left leg pain despite advanced imaging, such as magnetic resonance imaging (MRI), and orthopaedic input, which was focused onto her left knee being the prime cause of her pain. Her non-resolving symptoms prompted her to attend the Emergency Department. A careful clinical examination pointed towards the left proximal tibia being the most probable cause. Basic radiographic imaging (x-ray) of the left tibia revealed a lytic lesion which was later confirmed to be metastatic disease arising from a Pancoast tumour (PT) following further advanced imaging and diagnostics. This case highlights a unique presentation of a Pancoast tumour that, to our knowledge, has never been reported before in the medical literature. A high index of suspicion, careful examination, and investigation were essential to reach this diagnosis.

## Introduction

Pancoast tumours (PT) are apical tumours of the lung that manifest with a variety of symptoms. These tumours represent about 3% to 5% of all lung cancers [1]. Statistical data released by the World Cancer Research Fund (WCRF) estimates that about two million (12.8%) of all newly diagnosed cancers were lung cancers [2].

On average, these cancers often affect those over 60, and men are more affected than women. They are a clinically unique and challenging subset of non-small cell carcinoma of the lung (NSCLC). Classical symptoms are varied and include severe and persistent pain in the shoulder and arm, as well as in the distribution of the first and second thoracic nerve trunks and eighth cervical nerve trunk, Horner's syndrome, and weakness and atrophy of the muscles the hand [3]. 

Patients presenting with nonspecific or atraumatic pain are at risk of receiving suboptimal treatment in the emergency department (ED), leading to life-threatening conditions being missed and exposing the patient to greater risk. Each time a patient presents to a clinician, a clean slate approach should be adopted to prevent confirmation biases, particularly when appropriate imaging is not applied, leading to a missed or delayed diagnosis. This can ultimately lead to poor patient outcomes. This case pertains to the challenges of an unusual presentation of PT despite prior specialised review and magnetic resonance imaging (MRI). Accurate history-taking and physical examinations are the cornerstones of every doctor and patient interaction. Furthermore, diagnostics are only adjuncts. 

## Case presentation

A 56-year-old female, with a background history of hyperthyroidism, osteoporosis, oesophagitis, and hysterectomy, presented to the ED with a one-month history of severe left proximal tibial pain associated with weight-bearing which was worse at night with an insidious onset. Her history comprised of atraumatic pain without any signs or symptoms of infection and without any subjective weight loss or systemic features. She was an ex-smoker for 16 years. She had attended her general practitioner (GP) multiple times for the same pain over the preceding month. The GP organised an MRI of the left knee which was performed eight days before presentation to the ED. The MRI revealed a subcutaneous fat collection at the level of the left tibial tuberosity with no bony or ligamentous injury. Concerned about her symptoms, the patient attended a private orthopaedic consultant who, considering the MRI results, discharged her with simple analgesia. She subsequently decided to present to the ED for further clinical input due to worsening pain. 

On examination, her vital signs were normal. Systemically, she seemed well without any further symptoms. A focused examination of the left lower leg revealed mild erythema of the medial aspect of the proximal tibia without any swelling, bruising, or deformity. She had point tenderness over the medial aspect of the proximal tibia. Her gait was normal, and she had a good range of motion in her knee and ankle joint. Neurovascular examination of the lower limb was normal. No knee tenderness was elicited. The initial differential diagnoses considered were musculoskeletal pain/injury, stress fracture, gastrocnemius muscle tear/tightness, or fibromyalgia. Deep vein thrombosis (DVT), a common cause of lower limb pain was out ruled by a Wells' score of -2 and a D-dimer value of 0.29 mcg/mL (normal: < 0.44 mcg/mL).

The initial impression by the ED clinician was that the clinical area of concern was the tibia rather than the knee. However, the patient went on to have routine blood tests and an x-ray of the left tibia/fibula to further investigate the clinical examination findings elicited on the examination and persistent patient concerns.

Investigations 

Baseline blood tests were ordered and the results were all unremarkable (Table 1).

**Table 1:** Laboratory Results ALT: alanine aminotransferase; CRP: C-reactive protein; GGT: gamma-glutamyl transferase; WBC: white blood cells

Blood test	Result	Normal Range	Units
Renal Profile			
Sodium	140	135 - 145	mmol/L
Potassium	3.8	3.5 - 5.0	mmol/L
Urea	4.1	2.0 - 7.0	mmol/L
Creatinine	62	45 - 84	μmol/L
Liver Profile			
Total Protein	81	65 - 85	g/L
Albumin	45	35 - 50	g/L
Total Bilirubin	3	< 17	μmol/L
GGT	17	<40	U/L
Alkaline Phosphate	95	35 - 105	U/L
ALT	20	1-35	U/L
CRP	36	< 5	mg/dL
Bone Profile			
Calcium	2.42	2.15 - 2.55	mmol/L
Corrected Calcium	2.32	2.15 - 2.55	mmol/L
Albumin	45	35 - 50	g/L
Phosphate	1.20	0.80 - 1.40	mmol/
Haematology			
WBC	6.9	4.0 – 11.0	10^9^/L
Neutrophils	3.7	2.0 – 7.5	10^9^/L
D-Dimers	0.29	< 0.44	mcg/mL

**Figure 1:**
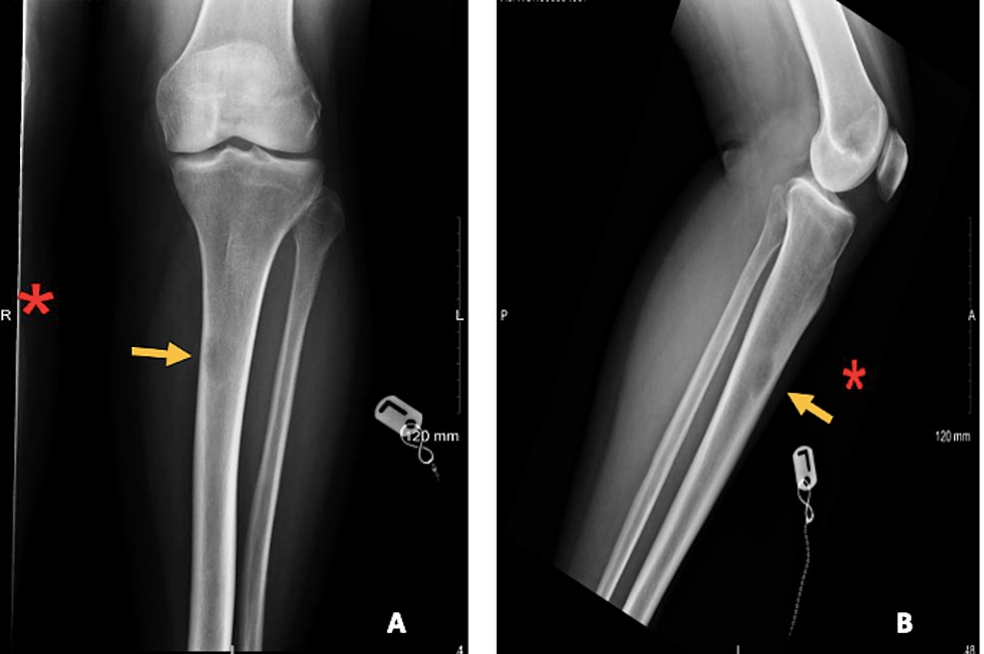
Left tibia/fibula radiography (A) Anteroposterior view of the left tibia/fibula radiography and (B) lateral view of the left tibia/fibula radiography show focal intramedullary lytic lesion at the proximal left tibia (arrow) suggestive of bone tumour.

**Figure 2:**
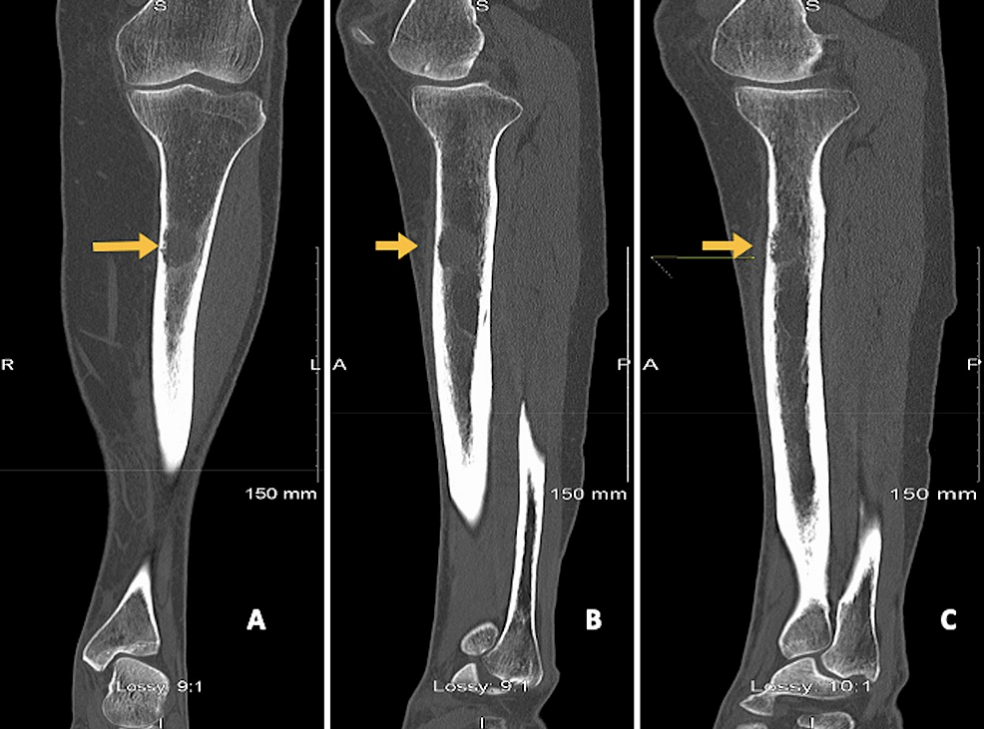
Computed tomography (CT) scan of the left tibia and fibula (A) Coronal non-contrast CT scan view of the left tibia and (B-C) sagittal non-contrast CT scan views of the left tibia showing a well-defined osteolytic tumour of the proximal shaft/diaphysis of the left tibia (arrow) seen with a moth-eaten appearance of the internal table of the cortical anteromedially. Appearances are suggestive of myeloma, metastatic deposit, or chondrosarcoma as the main differentials.

**Figure 3:**
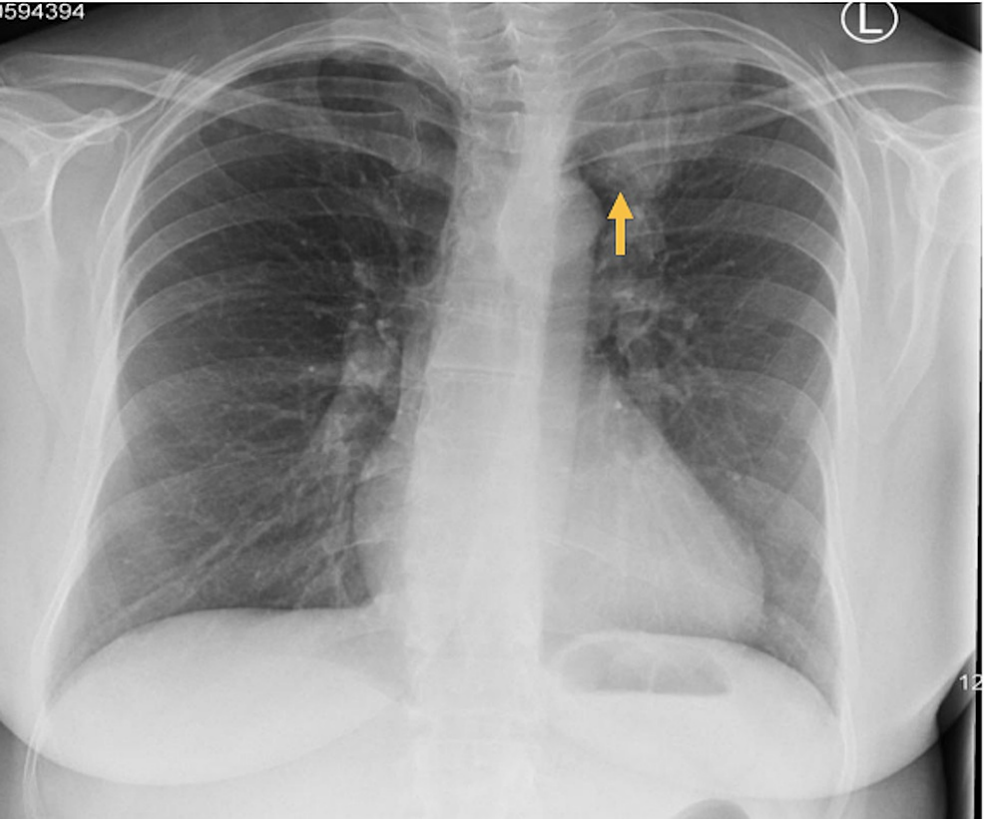
Chest x-ray showing opacification of left lung apex with slightly prominent upper left hilum (arrow)

**Figure 4:**
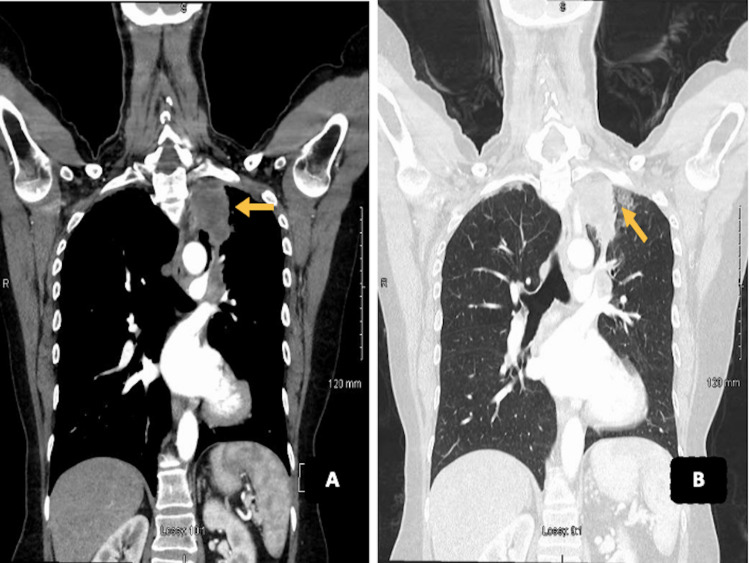
CT of the neck and thorax with contrast (A) Intravenous (IV) contrast-enhanced CT of the neck and thorax and (B) IV with multiplanar reconstruction showing a 5.4 x 4.1 x 6 cm left apical mass, which is associated with volume loss of the left upper lobe, suspicious for a Pancoast tumour.

A radiographic image of the left tibia showed an abnormal circular pattern approximately 12 cm below the knee (Figure 1). This incidental finding of a well-defined lytic lesion prompted further investigation with computed tomography (CT) scan of the lower leg which supported the possibility of metastatic disease of the bone (Figure 2). A chest x-ray was ordered for the staging of the disease as a baseline investigation. This demonstrated the presence of an apical lesion of the left lung apex, suggestive of metastatic bone disease from a possible primary lung cancer (Figure 3). A CT scan of the head/neck/chest with contrast was ordered to identify the source and staging of the disease (Figure 4). The CT scan of the chest confirmed the presence of an apical mass with no metastasis found in the abdomen, pelvis, or other parts of the chest. A multidisciplinary team approach involving the medical and orthopaedic teams was adopted in this case. A transbronchial needle aspiration (TBNA) was performed from the gland by the respiratory physicians and a sample was sent for cytology and histology. The results indicated a poorly differentiated non-small cell lung cancer (squamous) of the left lung apex with proven central adenopathy (T3N2Mx). Bronchoscopy didn’t yield any cytological evidence of the tumour cells. The primary lung lesion was visualized on endobronchial ultrasound (EBUS) which reported a 12 mm lymph gland in the left lower paratracheal area and a 15 mm lymph gland was noted at the superior left hilum. The multidisciplinary team recommended an urgent positron emission tomography (PET) scan to complete staging and liaison with medical oncology for consideration of systemic chemotherapy (± radiotherapy, depending on the final stage). The PET scan confirmed a 3 cm left proximal left tibial mass with avid uptake. 

Differential diagnosis

Chronic lower leg pain comprises several etiologies, such as stress fracture, chronic exertional compartment syndrome, medial tibial stress syndrome, nerve entrapment, peripheral vascular disease, DVT, and popliteal artery entrapment syndrome. Symptoms associated with these conditions often overlap, making a definitive diagnosis difficult [4]. There was little in this patient's history or physical examination to suggest any serious pathology. One of the differential diagnoses was cellulitis of the left upper leg, but the onset and lack of common features of cellulitis, such as redness, pain, swelling, and heat, made it very unlikely [5]. Necrotizing fasciitis is a diagnosis of choice if associated with a history of rapid progression when the pain is out of proportion to the clinical signs [6]. This patient’s symptoms, clinical examination, or laboratory data suggested neither cellulitis nor necrotising fasciitis.

Although DVT could have been among the differential diagnoses, there was no evidence of any other cardinal features of DVT, such as asymmetrical swelling in the extremity [7]. The two-level DVT Wells' score was also unlikely for DVT [8]. Most myopathic processes are relatively painless (polymyositis, dermatomyositis, toxic myopathies, and body myositis). Muscular pain is usually associated with trauma or repetition. Generally, muscle pain is confined to patients with acute rhabdomyolysis, a few metabolic myopathies, and myopathies associated with connective tissue disease [9]. Muscular pain was felt to be unlikely following examination. One might consider a diagnosis of a rupture of the distal musculotendinous junction of the medial head of the gastrocnemius muscle (tennis leg). This condition is often seen in athletes performing sudden acceleration and deceleration manoeuvres [10]. The patient denied any history of trauma, and the physical examination revealed localised pain at the medial aspect of the upper left leg. In stress fractures and medial tibial stress syndrome (MTSS) (known as shin splints), the patient presents with diffuse pain and tenderness over the posteromedial aspect of the distal tibia that occurs with exercise or at rest in more advanced cases [11]. Our patient’s left leg x-ray showed a lytic lesion of the proximal tibia without any fracture (Figure 1). Primary bony tumours or metastases are always considered when patients present with non-traumatic pain. Isolated leg pain is not commonly associated with a PT (lesions in the superior sulcus). Leg pain can direct towards malignancy in the presence of constitutional symptoms or red flags. In the absence of any history of cancer, red flags suggestive of malignancy are usually a new onset of severe pain in a patient over 60 years of age, non-mechanical pain, mechanical symptoms not responding to adequate treatment over an acceptable time, unexplained weight loss associated with fatigue, fever, and night sweats. Our patient’s presentation was atypical, but her perseverance was crucial in prompting further investigations. 

**Figure 5:**
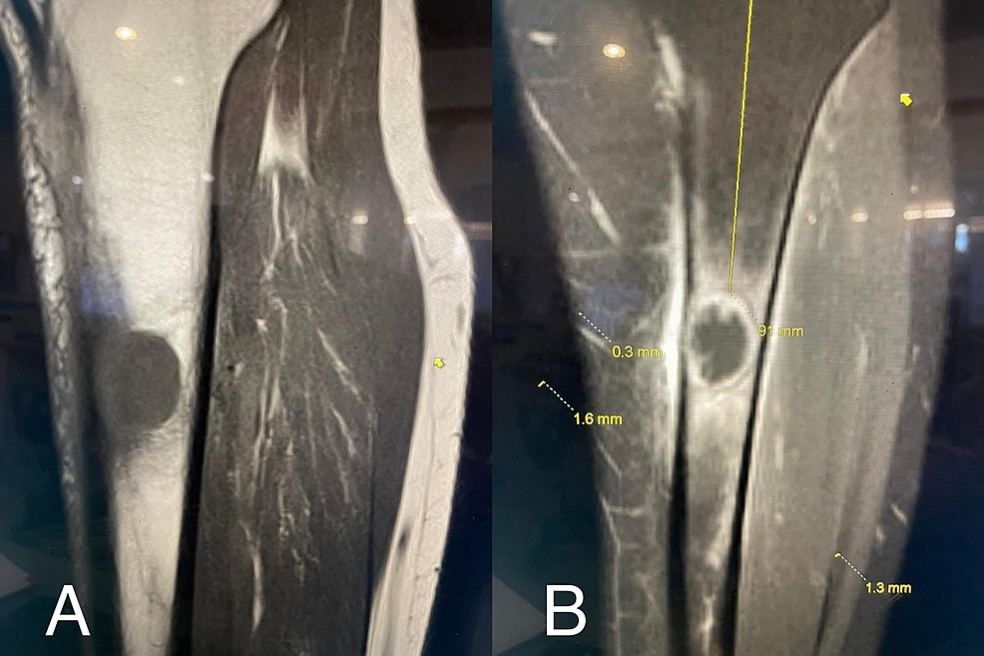
Magnetic resonance imaging (MRI) of the left tibia showing sagittal and coronal views (A) Sagittal view and (B) coronal view of the left tibia, confirming an osteolytic bony lesion noted in the upper shaft of the left tibia and with its superior aspect. Appearances are suggestive of an aggressive bony lesion.

It is common practice to obtain x-rays of the tibia and fibula in the presence of trauma or suspicion of any bony pathology. There were initially very few indications for obtaining such imaging on this patient. The x-ray of the left leg, in this case, was organised to rule out a stress fracture. The incidental finding of a well-defined lytic lesion prompted the physician to investigate further with a CT scan of the lower leg which supported the possibility of metastatic disease of the bone (Figures 1-2). A chest x-ray was obtained to assist with the staging of the disease. This demonstrated the presence of an apical lesion in the left lung apex, suggestive of metastatic bone disease from a possible primary lung cancer (Figure 3). A CT of the chest was ordered to confirm the existence of an apical mass and its relationships with the bony thorax and the thoracic inlet (Figure 4) [12]. To further assess our patient, an MRI of the left tibia was requested, which confirmed an osteolytic bony lesion noted in the upper shaft of the left tibia and with its superior aspect suggestive of an aggressive bony lesion (Figure 5). An MRI of the chest remained the best imaging modality to diagnose a superior sulcus tumour and it allowed for the proper assessment of possible neurological impairment [13-14].

Outcome and follow-up 

**Figure 6:**
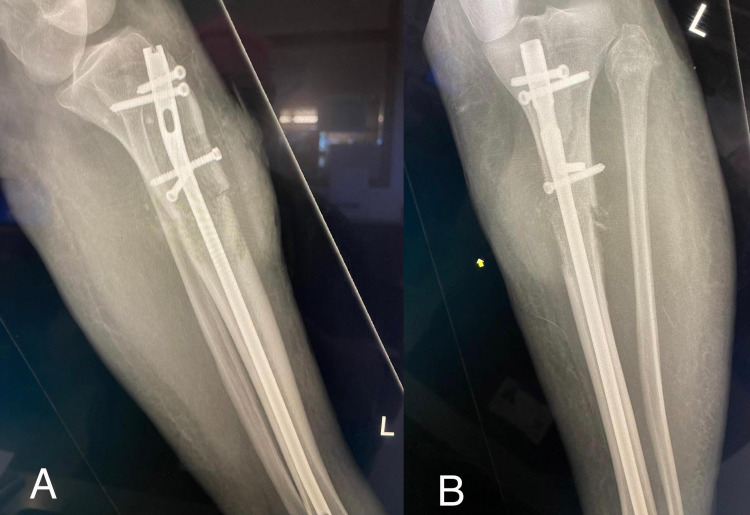
X-Ray left tibia and fibula (A-B) Lateral views of the left tibia and fibula radiography showing pathological transverse fracture of the proximal left tibial shaft post-internal fixation. The aggressive lytic lesion in the proximal left tibia appears slightly bigger as does the adjacent associated soft tissue mass with a satisfactory alignment post-internal fixation.

Unfortunately, her outcome was poor, and the patient developed multiple complications. A few months after the diagnosis, she presented to the ED again with respiratory sepsis post-radiotherapy and required admission to the Intensive Care Unit; this was successfully treated with intravenous (IV) antibiotics. A month following her ICU admission, she developed a pulmonary embolism in the right lung and was started on anticoagulants. She also suffered from a pathological fracture of the left proximal tibia which required surgical intervention (Figure 6). The patient went on to develop hoarseness due to tumour invasion of the recurrent laryngeal nerve and developed left arm severe pain, likely secondary to brachial plexus compression, confirmed with an interval scan due to increased tumour size.

After an informed and thorough discussion with the caretaker team and the patient, a decision was made in the patient’s best interests to refer her for palliative care. The case was consulted by a palliative medicine physician. She was admitted to the local hospice where she was started on therapeutic enoxaparin, 1 mg/kg twice a day, and alprazolam, methadone, and oxycodone for pain management. She received 10 sessions of palliative radiotherapy and benefited from neoadjuvant chemotherapy post-radiotherapy.

## Discussion

PTs represent only 3% to 5% of all lung cancers, similar to non-small cell lung cancers (NSCLC) with a predilection for distant metastasis [1]. The presentation of isolated leg pain as the sole sign of a PT is rare and not previously documented. In most cases, PTs present most commonly with shoulder and arm pain, Horner’s syndrome, and weakness and atrophy of the muscles of the hand. Uncommon symptoms, such as cough, haemoptysis, and dyspnoea, manifest late in the disease. The imaging was the key to unlock the dilemma of this patient’s longstanding deadly condition. 

Non-specific symptoms can be a sole indication of an underlying serious pathology and a high degree of clinical suspicion is advised for clinicians with similar cases. Comprehensive history taking, meticulous physical examination, and appropriate diagnostic imaging are pillars of confined and timely diagnosis and prognosis. The importance of these pillars cannot be highlighted enough as they are the cornerstone for clinicians for making an accurate diagnosis. 

Generally, laboratory investigations are unremarkable in PTs [15]. Given the anatomical location of the tumour, a bronchoscopic biopsy is less precise [16]. The diagnostic of choice remains an image-guided transthoracic needle aspiration biopsy or fine-needle aspiration, as in our case, with a 90% success rate. Core needle biopsy is generally preferred to provide tissue for histology. The cytology of the transbronchial needle aspiration from the gland and histopathology concluded a poorly differentiated NSCLC (squamous) in the left lung apex with proven central adenopathy (T3N2Mx).

Based on the existing Tumour, Node, and Metastasis (TNM) classification system, PTs are generally described as T3 lesions due to their unique location and involvement in the chest wall. T4 lesions involve disease progression to the brachial plexus, mediastinum, or vertebral bodies. N2 described the involvement of the ipsilateral mediastinal and/or subcarinal nodes. In the absence of these metastases, PTs are often classified as Stage IIB or Stage IIIA or IIIB [17]. M1 defines the presence of metastases in the ipsilateral lobe of non-primary lung tumour or affecting other organs [18]. Mx reports that metastasis could not be assessed. 

After appropriate diagnostic evaluation, surgical intervention remains the curative modality of choice, especially for patients with a restricted tumour of class T3N0M0. Although PTs were once considered to have a fatal prognosis, recent therapies (chemotherapy, radiotherapy, and immunotherapy), combined with resection, have improved the survival of patients [19]. Radiation therapy is often required to manage the pain [20]. With the most common site of the metastatic disease being the brain, prophylactic cranial irradiation of patients with PT is, therefore, recommended to prevent brain metastases. The five-year overall survival after preoperative radiotherapy associated with prolonged surgical resection is generally around 20% - 35%. With radiotherapy alone and without surgery, this rate ranges from 0 to 29% [17].

## Conclusions

Accurate history taking and a thorough physical examination are cornerstones of every doctor and patient interaction. This case is a rare and undocumented presentation of a PT, particularly in the absence of constitutional symptoms or typical features of a PT. Having a holistic approach by the physicians is vital for considering alternative presentations. Meticulous clinical examination and appropriate investigations are necessary to enable diagnosis. Patient insistence and symptomatology should always be considered seriously as it might lead the clinicians towards the correct diagnosis and minimise confirmation bias. Pain out of proportion is always a red flag symptom for a more sinister pathology. 

## References

[1] Detterbeck FC (1997). Pancoast (superior sulcus) tumors. Ann Thorac Surg.

[2] Gundepalli SG, Tadi P (2021). Lung Pancoast Tumor. StatPearls [Internet].

[3] Jett JR (2003). Superior sulcus tumors and Pancoast’s syndrome. Lung Cancer.

[4] Edwards PH Jr, Wright ML, Hartman JF (2005). A practical approach for the differential diagnosis of chronic leg pain in the athlete. Am J Sports Med.

[5] Sullivan T, de Barra E (2018). Diagnosis and management of cellulitis. Clin Med (Lond).

[6] Borschitz T, Schlicht S, Siegel E, Hanke E, von Stebut E (2015). Improvement of a clinical score for necrotizing fasciitis: 'pain out of proportion' and high CRP levels aid the diagnosis. PLoS One.

[7] Stone J, Hangge P, Albadawi H (2017). Deep vein thrombosis: pathogenesis, diagnosis, and medical management. Cardiovasc Diagn Ther.

[8] (2021). Venous thromboembolism overview. http://pathways.nice.org.uk/pathways/venous-thromboembolism.

[9] Seward B Rutkove (2021). Differential diagnosis of peripheral nerve and muscle disease. UpToDate.

[10] Chen CP, Tang SF, Hsu CC, Chen RL, Hsu RCh, Wu CW, Chen MJ (2009). A novel approach to sonographic examination in a patient with a calf muscle tear: a case report. J Med Case Rep.

[11] Becker JA, Richardson BM, Brown ST (2016). A step-wise approach to exertional leg pain: this review, differential table, and case to test your skills can help you avoid overuse of costly tests and delayed treatment.. J Fam Pract.

[12] Marulli G, Battistella L, Mammana M, Calabrese F, Rea F (2016). Superior sulcus tumors (Pancoast tumors). Ann Transl Med.

[13] Rusch VW (2006). Management of Pancoast tumours. Lancet Oncol.

[14] Fontinele e Silva J, Barbosa Mde P, Viegas CL (2009). Small cell carcinoma in Pancoast syndrome. J Bras Pneumol.

[15] Karl J D'Silva (2021). Pancoast Syndrome Workup: Approach Considerations. http://emedicine.medscape.com/article/284011-workup.

[16] Arcasoy SM, Schild SE (2020). Superior pulmonary sulcus (Pancoast) tumors. UpToDate.

[17] Arcasoy SM, Jett JR (1997). Superior pulmonary sulcus tumors and Pancoast's syndrome. N Engl J Med.

[18] Deslauriers J, Grégoire J (2000). Clinical and surgical staging of non-small cell lung cancer. Chest.

[19] International Association for the Study of Lung Cancer Staging Project (2016). IASLC Staging Handbook in Thoracic Oncology, Second Edition. North Fort Myers, FL: Editorial Rx Press.

[20] Heelan RT, Demas BE, Caravelli JF (1989). Superior sulcus tumors: CT and MR imaging. Radiology.

